# The potential role of human islet amyloid polypeptide in type 2 diabetes mellitus and Alzheimer’s diseases

**DOI:** 10.1186/s13098-023-01082-1

**Published:** 2023-05-13

**Authors:** Mohammed Alrouji, Hayder M. Al-Kuraishy, Ali I. Al-Gareeb, Athanasios Alexiou, Marios Papadakis, Hebatallah M. Saad, Gaber El-Saber Batiha

**Affiliations:** 1 Department of Clinical Laboratory Sciences, College of Applied Medical Sciences, Shaqra University, Shaqra, 11961 Saudi Arabia; 2 Department of clinical pharmacology and therapeutic medicine, college of medicine, ALmustansiriyiah University, M.B.Ch.B, FRCP, Baghdad, Box 14132, Iraq; 3 Department of Science and Engineering, Novel Global Community Educational Foundation, Hebersham, NSW 2770 Australia; 4 AFNP Med, Wien, 1030 Austria; 5 Department of Surgery II, University Hospital Witten-Herdecke, Heusnerstrasse 40, 42283 Wuppertal, Germany; 6 Department of Pathology, Faculty of Veterinary Medicine, Matrouh University, Marsa Matrouh, 51744 Egypt; 7 Department of Pharmacology and Therapeutics, Faculty of Veterinary Medicine, Damanhour University, Damanhour, 22511 AlBeheira Egypt

**Keywords:** Human islet amyloid polypeptide insulin resistance, Type 2 diabetes mellitus, Alzheimer’s disease

## Abstract

Human Islet amyloid polypeptide (hIAPP) from pancreatic β cells in the islet of Langerhans has different physiological functions including inhibiting the release of insulin and glucagon. Type 2 diabetes mellitus (T2DM) is an endocrine disorder due to relative insulin insufficiency and insulin resistance (IR) is associated with increased circulating hIAPP. Remarkably, hIAPP has structural similarity with amyloid beta (Aβ) and can engage in the pathogenesis of T2DM and Alzheimer’s disease (AD). Therefore, the present review aimed to elucidate how hIAPP acts as a link between T2DM and AD. IR, aging and low β cell mass increase expression of hIAPP which binds cell membrane leading to the aberrant release of Ca^2+^ and activation of the proteolytic enzymes leading to a series of events causing loss of β cells. Peripheral hIAPP plays a major role in the pathogenesis of AD, and high circulating hIAPP level increase AD risk in T2DM patients. However, there is no hard evidence for the role of brain-derived hIAPP in the pathogenesis of AD. Nevertheless, oxidative stress, mitochondrial dysfunction, chaperon-mediated autophagy, heparan sulfate proteoglycan (HSPG), immune response, and zinc homeostasis in T2DM could be the possible mechanisms for the induction of the aggregation of hIAPP which increase AD risk. In conclusion, increasing hIAPP circulating levels in T2DM patients predispose them to the development and progression of AD. Dipeptidyl peptidase 4 (DPP4) inhibitors and glucagon-like peptide-1 (GLP-1) agonists attenuate AD in T2DM by inhibiting expression and deposition of hIAP.

## Introduction

**Fig. 1:**
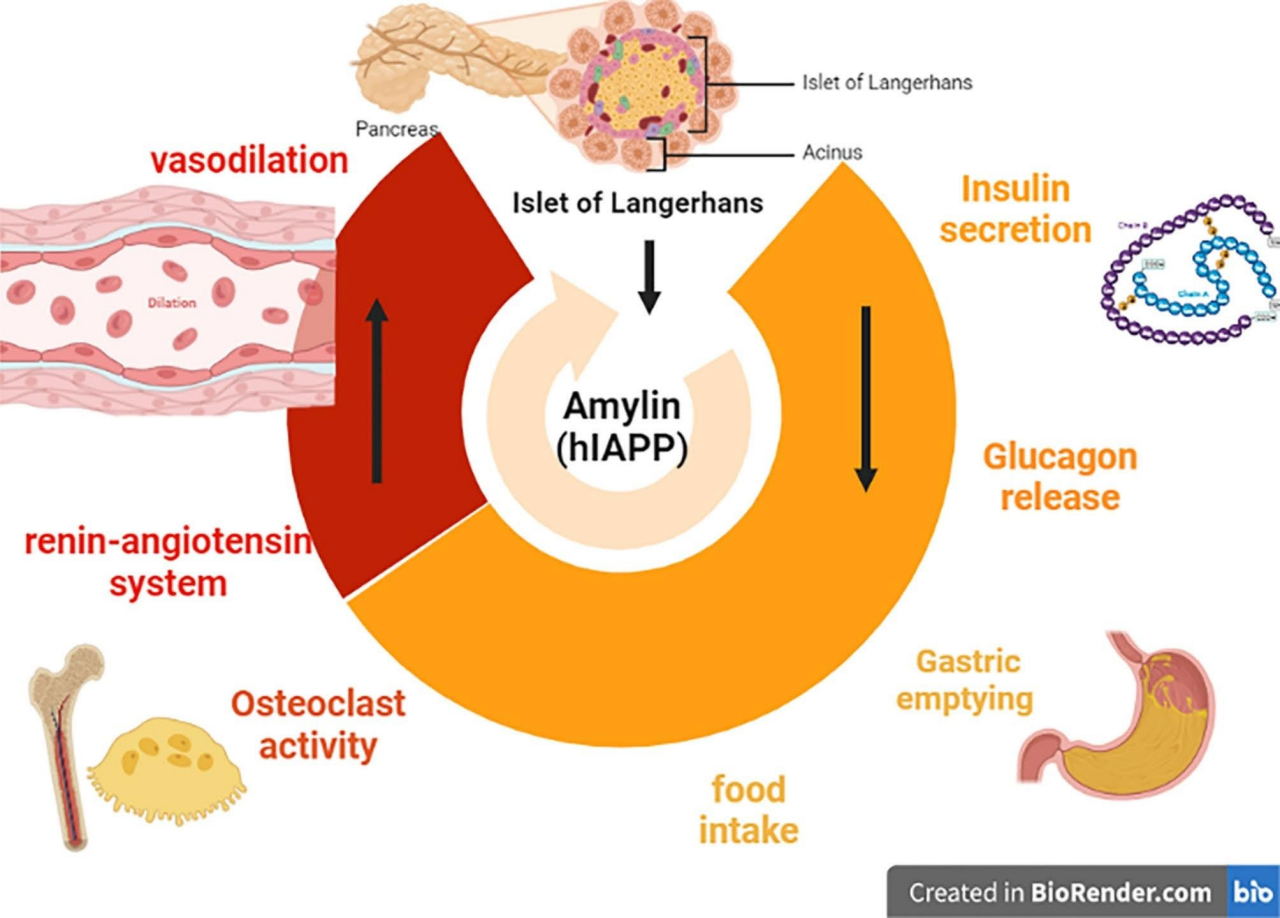
The physiological role of human Islet amyloid polypeptide (amylin)

Human Islet amyloid polypeptide (hIAPP) also called amylin is one of the main secretory products of pancreatic β cells in the islet of Langerhans [1]. hIAPP has different physiological functions including inhibiting gastric emptying, regulation of satiety and inhibiting the release of insulin and glucagon [1]. In addition, hIAPP has other effects including inhibition of bone resorption, vasodilation and regulation of the renin-angiotensin system (RAS) (Fig. 1) [1, 2].

hIAPP is co-secreted with insulin from pancreatic β cells, and due to its ability to aggregate into pancreatic islet cells in type 2 diabetes mellitus (T2DM) so-called hIAPP [2]. Aggregation of hIAPP induces cytotoxic effects leading to the loss of pancreatic β cells by inducing apoptosis with subsequent failure of pancreatic β cells and development of T2DM [3].

Notably, hIAPP was initially described in 1901 by weichselbam et al. and Opie et al. as an islet amyloid called islet hyalinization in patients with T2DM [4]. However, islet hyalinization is not specific to T2DM as it is present even in non-diabetic patients [5]. Cohen and Calkins [6] described the fibrous components of different amyloid and islet hyalinization. From these studies, it was concluded that islet hyalinization is part of amyloid proteins. Later on, in 1972 Pearse and his colleagues believed that islet amyloid fibrils might be a product of insulin or proinsulin [7]. In 1986, Westermark et al. described a novel peptide related to the calcitonin gene-related peptide (CGRP) in the pancreatic β cells extracted from insulinoma which was named an insulinoma amyloid peptide [8]. This peptide was renamed as a diabetes-associated peptide in 1987 as hIAPP or amylin later on [9, 10]. Of note, hIAPP is a 37 amino acid hormone co-released and co-stored with insulin and shared a similar enzymatic process with other hormones like CGRP, calcitonin and adrenomedullin [11]. It has been shown that hIAPP is released in a pulsatile manner similar to insulin and circulated in glycosylated and non-glycosylated forms. In healthy subjects, hIAPP circulated levels ranged from 4-25pmol/l, distributed equally with insulin [1, 12]. Unlike insulin which is eliminated by the liver, hIAPP is eliminated by renal metabolism [1]. As well, unlike the insulin gene which is located on chromosome 11, the hIAPP gene is located on chromosome 12 [13]. Pre-pro-hIAPP in the endoplasmic reticulum is converted to pro-hIAPP in the secretory vesicles [14]. Both pro-hIAPP and pro-insulin are processed by endoproteases in the Golgi and secretory granules in a PH-dependent manner [1].

Expression of hIAPP is not limited to the pancreatic β cells but is also expressed on α cells of the islet [11]. In addition, hIAPP is expressed in sensory neurons, the large intestine, the antrum, the brain, and the pancreas [11]. It has been shown that proinsulin and insulin prevent aggregation of hIAPP to form amyloid [15]. Insulin is regarded as a potent inhibitor of hIAPP aggregation, thus insulin resistance (IR) promotes aggregation of hIAPP to form amyloid fibril in the pancreatic β cells [16]. Since, hIAPP is co-released with insulin, though plasma concentration of hIAPP represents 1–2% of that of insulin [16, 17]. Following glucose stimulation, plasma hIAPP is stimulated and increased in parallel with that of insulin [17]. hIAPP is mainly metabolized by the insulin-degrading enzyme (IDE) and neprilysin (NEP) [18, 19]. IDE is a Zinc-metalloprotease expressed in different cells involved in the degradation of hIAPP and amyloid beta (Aβ) preventing hIAPP and Aβ-induced cytotoxicity [18]. NEP is a Zinc-metalloprotease degraded both of hIAPP and Aβ [18, 20]. NEP is highly expressed in pancreatic β cells however; its expression is reduced by aging [18]. Of interest, hIAPP acts on the specific receptors called receptor activity modifying proteins (RAMPs) which are expressed in the brain and renal cortex [20]. Remarkably, hIAPP has structural similarity with Aβ and can involve in the pathogenesis of T2DM [21] and AD [22]. Therefore, the present review aimed to elucidate how hIAPP acts as a link between T2DM and AD.

## Role of hIAPP in T2DM

T2DM is an endocrine disorder due to relative insulin insufficiency and IR, characterized by hyperglycemia and cardiometabolic complications [22]. T2DM represents 95% of all diabetes types and contributes to 5% of all mortalities under the age of 70 [23, 24]. Genetic, epigenetic, environmental factors, obesity, smoking and male sex are the main risk factors involved in the development and progression of T2DM [25, 26]. Particularly, aggregation of hIAPP is associated with the development of T2DM by inducing apoptosis and loss of pancreatic β cells in the islet of Langerhans [21]. However, hIAPP had been reported to have a protective effect against the degeneration of pancreatic β cells in mice with alloxan-induced diabetes [27]. The protective effect of hIAPP on the pancreatic β cells is through the improvement of pancreatic microcirculation and limit hyperpolarization of β cells [27]. A case-control study involved diabetic patients and healthy controls showed that plasma hIAPP was lower than insulin in healthy subjects; it increased in obese subjects and was undetectable in T1DM. In T2DM obese patients, plasma hIAPP level was similar to healthy controls but low in T2DM non-obese patients, suggesting the role of hIAPP with insulin in the regulation of glucose homeostasis [28].

Deficiency of IDE which is involved in the degradation of hIAPP induces the development of hyperamylinemia which triggers the development of IR [29]. In vitro study demonstrated that inhibition of IDE increases the risk of hIAPP-induced cytotoxicity [30]. However, acute administration of IDE inhibitor leads to beneficial effects on glucose tolerance in mice by increasing hIAPP which decreases gastric empty time [31]. Different studies illustrated that chronic use of IDE inhibitors leads to metabolic dysfunction via dysregulation of the proteasome pathway and chaperon-mediated autophagy [30, 32]. In vitro study demonstrated that inhibition of IDE by bacitracin increases the risk for the development of hIAPP-induced cytotoxicity through inhibition clearance of hIAPP [30]. In contrast, inhibition of IDE may improve glucose homeostasis [32].

**Fig. 2:**
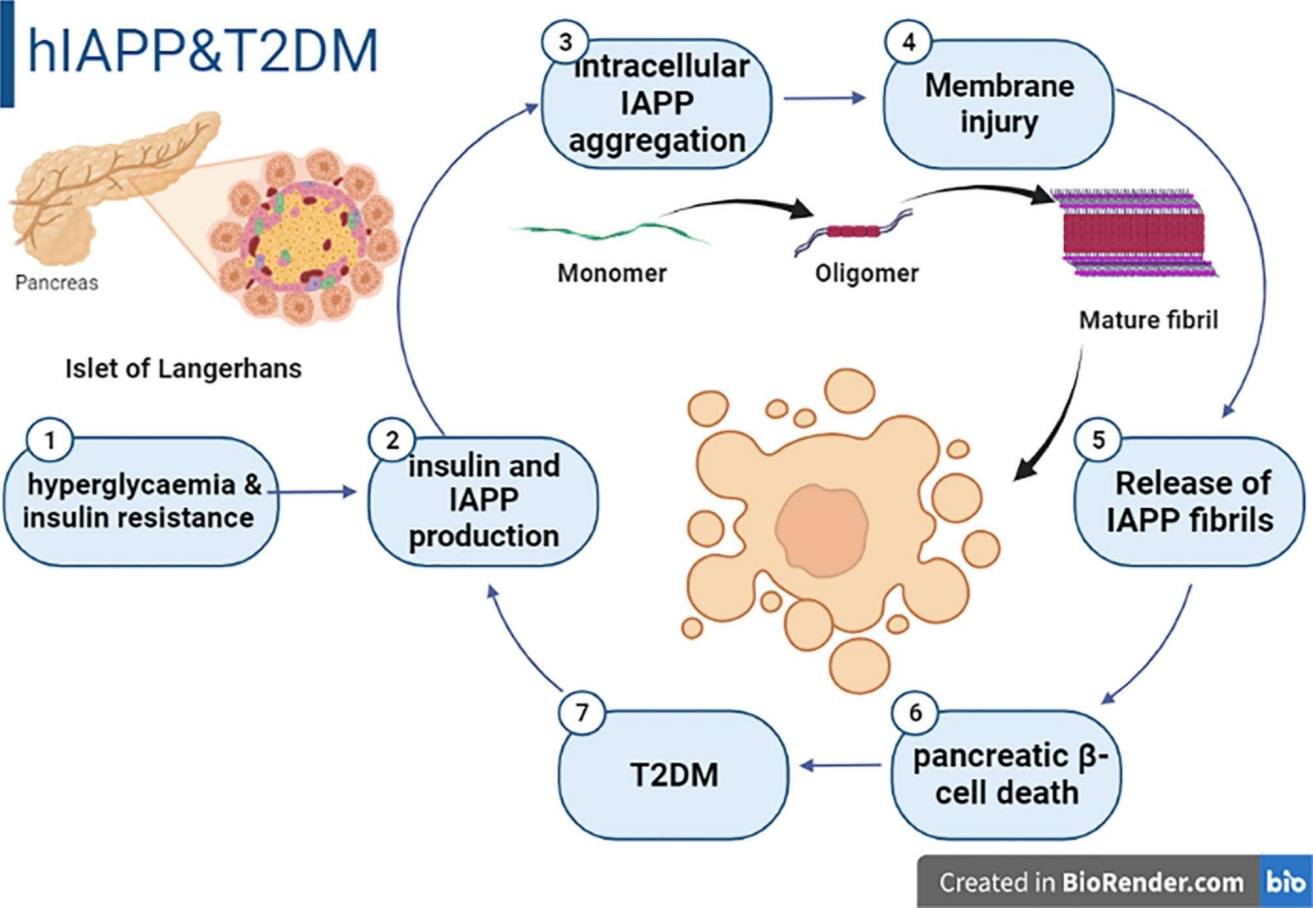
Human Islet amyloid polypeptide (hIAPP) and type 2 diabetes mellitus (T2DM)

Notably, hIAPP unlike rat IAPP is highly subjected to amyloidogenesis by forming amyloid fibril. The amyloidogenic propensity of hIAPP is related to the presence of 20–29 and 8–18 residues which have no proline as in rat IAPP as proline prevents aggregation of hIAPP [33]. Higher level of plasma hIAPP in prediabetic and diabetic patients promotes the formation of hIAPP protofibrils leading to progressive dysfunction of pancreatic β cells with the development of T2DM which in turn promote the secretion of hIAPP in a vicious cycle (Fig. 2) [33].

In addition, a monoclonal antibody to hIAPP could be an early diagnostic biomarker for T2DM diagnosis [33]. These findings raised an intricate question is hIAPP represents dysfunction of pancreatic β cells or is it an etiological factor in the development of T2DM? Clark et al. [34] suggested that the deposition of hIAPP and formation of pancreatic β cells amyloidosis is a consequence of T2DM since in humans T2DM islet amyloidosis is present in less than 1% to more than 80%. Extensive deposition of hIAPP is correlated with the reduction of dysfunction of pancreatic β cells in animal and human studies [35, 36]. Islet amyloidosis is not developed in T1DM where both insulin and hIAPP are absent [34]. Bram et al. [37] observed that hIAPP-induced pancreatic β cell apoptosis was prevented by diabetes-associated antibodies. However, glucose intolerance and hyperglycemia promote oligomerization of hIAPP [34]. Once protofibril is formed, it acts as nucleation nidus promoting progressive fibril generation and development of IR [34]. Remarkably, there is a vicious cycle between hIAPP and glycated insulin through exacerbation of pancreatic β cytotoxicity in T2DM [38]. Notoriously, sulfonylurea promotes fibril formations in diabetic cats [39]. ATP-sensitive potassium channel, a site activated by sulfonylurea, is required for hIAPP to induce pancreatic β cells dysfunction [40, 41].

Therefore, the oligomerization of hIAPP is controlled by the balance between processing and degradation pathways [42]. Genetic variants of these pathways affect the prevalence of T2DM in the Chines population [42]. Other factors are also involved in enhancing the oligomerization of hIAPP to amyloid fibrils and the formation of amyloid in the pancreatic β cells [43]. Not all forms of hIAPP aggregates induce the destruction and apoptosis of pancreatic β cells, though only toxic hIAPP aggregates can cause these events [43].

### The underlying mechanisms that increase hIAPP aggregates

It has been shown that the incidence and prevalence of T2DM are increased with age due to increase aggregation of hIAPP and the development of pancreatic β cells [44]. Of interest, hIAPP and associated monomers have normal biological activity on normal pancreatic β cells [45]. However, toxic oligomers lead to membrane toxicity in T2DM [45]. Toxic non-soluble fibrils from hIAPP interact with lipids in the cell membrane disrupting negative charge phospholipid [45]. Besides, the hydrophobic part of non-soluble fibrils induces protein misfolding at acidic PH [46].

The underlying causative factors that convert soluble hIAPP oligomers to toxic non-soluble fibrils are largely unknown [47]. Nevertheless, different environmental factors may induce mutations like the S20G mutation which result in more formation of the toxic non-soluble fibrils [47]. As well, a single mutation in F15 of hIAPP oligomers increases the biosynthesis of toxic non-soluble fibrils [48]. These mutations affect the solubility of hIAPP oligomers by changing the sequence of proline [49]. In addition, abnormal processing of hIAPP may promote the formation of toxic non-soluble fibrils [48, 49]. The absence of proprotein convertase 2 which involves the processing and removal of hIAPP oligomers enhances the generation of toxic non-soluble fibrils and induction dysfunction of pancreatic β cells [49]. In addition, the dysfunction of the deamidation process which is involved in the stability, structure, and aggregation of hIAPP oligomers by adding a negative charge promotes the production of unmodified hIAPP oligomers and induces the formation of toxic non-soluble fibrils [50]. Abnormality in the disulfide bonds C2-C7 increases the formation of toxic non-soluble fibrils by affecting the kinetic process of hIAPP oligomers [51]. It has been shown that copper ions encourage the aggregation of hIAPP oligomers by inducing reactive oxygen species (ROS) [52]. Copper chelating agents can reduce T2DM risk by reducing the formation of ROS and the production of unmodified hIAPP oligomers [52]. Notoriously, heparin plays a critical role in the production of hIAPP oligomers and the formation of toxic non-soluble fibrils [53]. The negative charge of heparin binds the positive charge in the N-terminal of hIAPP oligomers. This binding enhances hIAPP oligomers to be converted to toxic non-soluble fibrils and attenuates hIAPP-induced cytotoxicity [53].

### Mechanisms of hIAPP oligomers-induced cytotoxicity

**Fig. 3:**
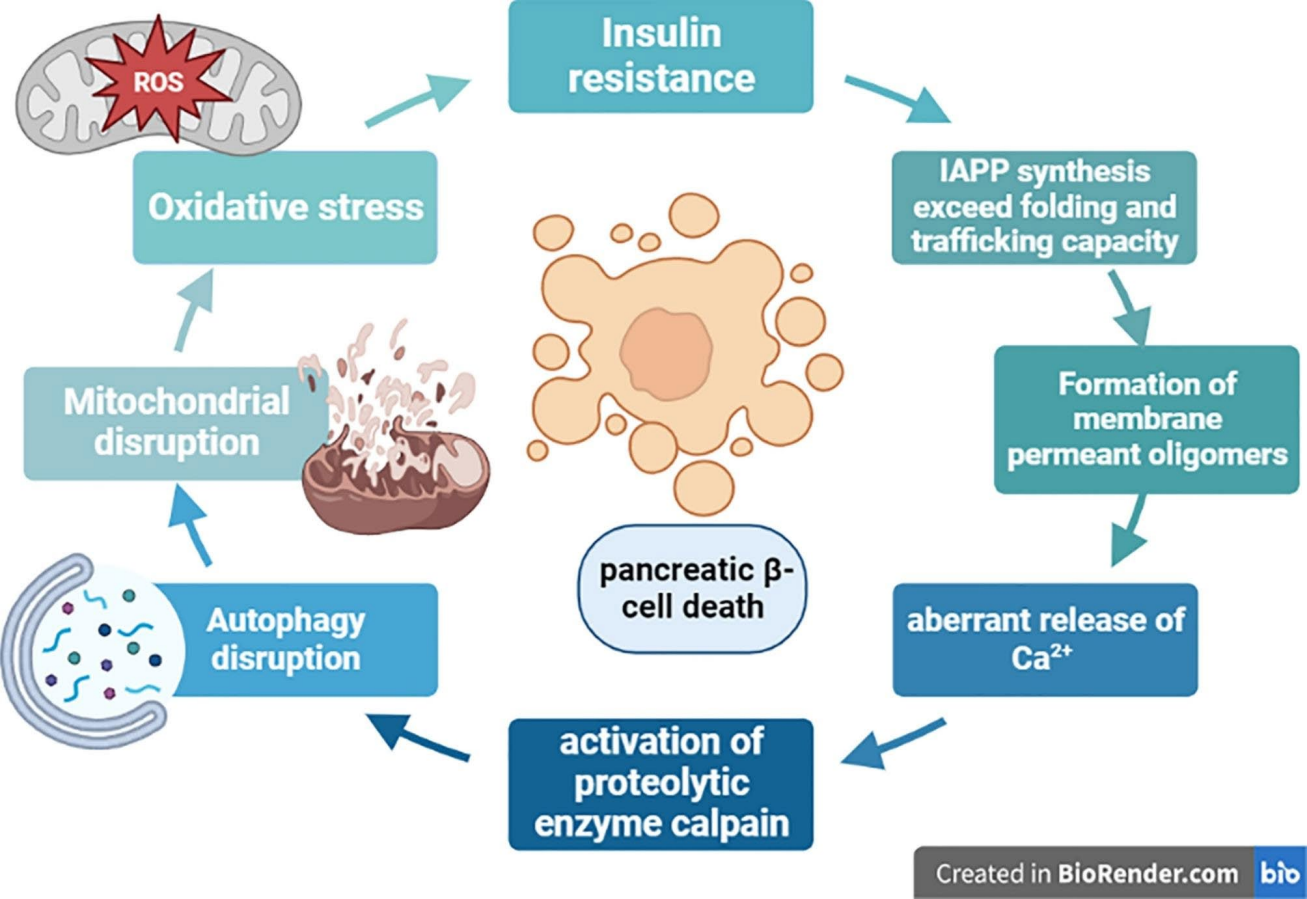
Human Islet amyloid polypeptide (hIAPP) and loss of β cells

Monomeric and oligomeric hIAPP disrupt cell membrane fluidity by increasing the production of ROS causing membrane leakage and cytotoxicity [54]. Monomeric hIAPP can interact with different proteins leading to cytotoxicity [54]. A higher concentration of hIAPP is subjected to form aggregation [55]. For example, the higher concentration of hIAPP in IR enhances its aggregation with the formation of toxic non-soluble fibrils [55]. Furthermore, the higher concentrations of hIAPP and aggregated fibrils can induce endoplasmic reticulum (ER) stress which are the hallmark of T2DM [56]. In turn, oxidative stress, ER stress, and hyperglycemia promote the aggregation of toxic non-soluble fibrils [56]. Markedly, IR, aging, and low β cell mass increase expression of hIAPP which binds cell membrane leading to the aberrant release of Ca^2+^ and activation of proteolytic enzyme calpain leading to a series of events causing loss of β cells [57] **(**Fig. 3**)**.

In response to oxidative stress and ER stress, chaperon-mediated autophagy is upregulated to prevent cell injury [58]. Chaperon-mediated autophagy can bind hIAPP and prevent its aggregation [58]. It has been shown that impairment of autophagy contributes to the accumulation of toxic non-soluble fibrils and the development of dysfunction of pancreatic β cells [58]. Therefore, restoration of autophagy and proteostasis of hIAPP may prevent hIAPP-induced cytotoxicity. In T2DM patients, the serum level of chaperone heat shock protein 70 (Hsp70) is increased [59]. A case-control study included 36 newly diagnosed T2DM patients, 37 long-standing T2DM patients and 36 healthy controls showed that chaperon Hsp70 serum level was higher in T2DM patients and correlated with the disease duration compared to the controls [59]. Ladjimi et al. [60] observed that chaperon Hsp70 in T2DM is augmented to inhibit aggregation of hIAPP. Therefore, increased chaperone Hsp70 serum level could be a compensatory mechanism against the development of oxidative stress and ER stress in T2DM. Augmentation of endogenous or use of exogenous chaperon Hsp70 activators could be effective in the attenuation of hIAPP-mediated pancreatic β cytotoxicity [60]. Administration of alfalfa-derived Hsp70 in patients with IR could be effective in the prevention of T2DM [60].

Furthermore, heparan sulfate proteoglycan (HSPG) can increase hIAPP aggregation [61]. Perlecan is a secretory part of HSPG that can bind hIAPP and increase its aggregation and cytotoxicity as confirmed in vitro and in vivo [61]. Loss of perlecan in transgenic mice prevents the accumulation of hIAPP and maintains glucose homeostasis [61]. In the components of islet amyloid aggregates, there is also serum amyloid protein, apolipoprotein E4 and HSPG which increase the aggregation of hIAPP [61]. Perlecan which is localized in the pericapillary matrix of the islet is mainly involved in the aggregation of hIAPP [61].

Moreover, the immune response to the formed hIAPP fibrils triggers the release of pro-inflammatory cytokines mainly IL-1β which increases hIAPP fibrils-induced β cytotoxicity and development of IR and T2DM [62]. In vitro study demonstrated that hIAPP activates toll-like receptor 2 (TLR2) with activation expression of nuclear factor kappa B (NF-κB) and induces the expression of pro-inflammatory cytokines [63]. This finding suggests a critical role of pro-inflammatory cytokines in the development and progression of inflammation in the pancreatic islet with the development of IR. Westwell-Westwell-Roper et al. [64] found that blocking IL-1β can attenuate hIAPP-induced cytotoxicity and pancreatic β cell dysfunction. The host defense mechanism against hIAPP induces the release of immunomodulatory cathelicidin which suppress the assembly of hIAPP and the associated injury of pancreatic β cell [65]. Thus, the immune response against hIAPP has bidirectional effects that could be harmful or beneficial.

Indeed, zinc prevents protein misfolding and aggregation of hIAPP [66]. Zinc deficiency enhances the aggregation of hIAPP and the development of injury of pancreatic β cells [66]. In vitro study demonstrated that zinc can bind un-aggregated hIAPP at a micro-microlar concentration [66]. Of interest, the mutation of zinc ion transporter on the secretory granules reduces hIAPP aggregation [67]. Brender et al. [68] illustrated that zinc has an inhibitory effect on the aggregation of hIAPP by increasing lag-time for hIAPP aggregation and interrupting the formation of toxic non-soluble fibrils. Zinc level is reduced in T2DM patients compared to healthy controls as documented in a case-control study [68]. Zinc deficiency in T2DM patients is associated with poor glycemic control [69] and this may explain why zinc deficiency enhances hIAPP aggregation and the associated dysfunction of pancreatic β cells.

In sum, hIAPP-induced dysfunction of pancreatic β cells through induction of oxidative stress and ER stress. Zinc deficiency and exaggerated immune response against hIAPP aggregation trigger more injury of pancreatic β cells.

## Role of hIAPP in AD

AD is the most common type of dementia characterized by progressive neurodegeneration leading to memory and cognitive deficits [70]. The pathological hallmarks of AD neuropathology are the deposition of extracellular Aβ and intracellular aggregation of tau protein which form neurofibrillary tangles (NFTs) [71]. AD affects more than 50 million individuals worldwide. One-third of AD cases are attributed to modifiable risk factors including obesity and T2DM [72]. It has been reported that the development of AD is linked to the duration and severity of T2DM [73].

T2DM patients have been reported to hold a higher incidence of cognitive decline and AD; T2DM has been intensely associated with an increased risk of developing all types of dementia, including AD [74]. A systematic review including 14 longitudinal population-based studies found that the incidence of any dementia was higher in T2DM patients than in those without T2DM [75]. Some studies have relied on the self-reported diagnosis of T2DM, and in the elderly population, many patients with T2DM may remain undiagnosed [76]. In a longitudinal cohort study, lasting up to 9 years, the risk of developing AD was 65% higher in T2DM patients as compared to the non-diabetic controls [77]. In a community-based controlled study, the prevalence of T2DM and glucose intolerance was examined in patients with AD vs. control participants without AD. The study suggested that T2DM was 35% and glucose intolerance was 46% might be present in up to 80% of patients with AD [78]. It has been shown that the incidence of dementia was higher in subjects with diabetes (14.9%) than that in nondiabetic subjects (10.3%) during the examination period between 1992 and 1999, with hazard ratio of 1.62 for AD in subjects with T2DM [78].

Disturbances in brain insulin signaling mechanisms represent early and progressive abnormalities and could account for the majority of molecular, biochemical, and histopathological lesions in AD [79]. Increasing IR and hyperinsulinemia were linked with more hippocampal and amygdale atrophy on magnetic resonance imaging in T2DM patients when compared to matched controls, regardless of vascular pathology [80]. It has been suggested that may be a common underlying mechanism predisposes to amyloid deposition in the brain and in the pancreatic islet [78].

Amyloid formation is a hallmark in both AD and T2DM due to the deposition of hIAPP in both pancreatic β islets and the brain [61]. Pancreatic-derived hIAPP can cross the blood-brain barrier (BBB). Besides, brain-derived hIAPP can interact with Aβ leading to progressive neuronal injury [81]. Brain and pancreatic hIAPP increase Aβ misfolding which are involved in AD neuropathology [82, 83]. Aggregation of hIAPP a pathological hallmark of T2DM is also observed in AD. Both hIAPP and Aβ exert similar cytotoxic mechanisms and share common physiochemical properties [83]. Similar to Aβ deposition in AD, hIAPP also aggregates in patients with T2DM to form pancreatic islet amyloid. Further, hIAPP and Aβ have a total of 25% amino acid sequence identity with high binding affinity to each other. They also share many biophysical and physiological properties and exert similar cytotoxic mechanisms when they aggregate. Furthermore, IAPP deposits have been found in the brain tissue of patients with AD, contributing to the pathophysiology of the disease [83]. These facts provide the basis to hypothesize that aggregation of amyloidogenic IAPP during T2DM plays a key role in the pathogenesis of AD [84]. The cross-seeding Aβ–hIAPP assemblies showed a wide range of polymorphic structures *via* a combination of four β-sheet-to-β-sheet interfaces and two packing orientations, focusing on a comparison of different matches of β-sheet layers [83]. Two cross-seeding Aβ–hIAPP assemblies with different interfacial β-sheet packings exhibited high structural stability and favorable interfacial interactions in both oligomeric and fibrillar states [83]. Both Aβ–hIAPP assemblies displayed interfacial dehydration to different extents, which in turn promoted Aβ–hIAPP association depending on interfacial polarity and geometry [84, 85]. Inflammation in the brain and pancreas is associated with cell degeneration and pathogenesis of both AD and T2DM. Inflammatory cascades in both tissues are triggered by the uptake of Aβ or IAPP aggregates by microglial cells or macrophages and their insufficient lysosomal degradation. This results in lysosomal damage, caspase-1/node like receptor pyrin 3 (NLRP3) inflammasome activation and release of IL-1β, a key pro-inflammatory cytokine in both diseases [83]. The inflammatory processes mediated by Aβ and IAPP aggregates are blocked by IAPP-GI, a non-amyloidogenic IAPP mimic, which forms high-affinity soluble and nonfibrillar hetero-oligomers with both polypeptides [83]. In contrast to fibrillar Aβ aggregates, nonfibrillar Aβ/IAPP-GI or Aβ/IAPP hetero-oligomers become rapidly internalized by microglial cells and targeted to lysosomes where Aβ is fully degraded. Internalization occurs via IAPP receptor-mediated endocytosis. In contrast to IAPP aggregates, IAPP/IAPP-GI hetero-oligomers become rapidly internalized and degraded in the lysosomal compartments of macrophages. Therefore, IAPP/Aβ cross-amyloid interaction and suggest that conversion of Aβ or IAPP into lysosome-targeted and easily degradable hetero-oligomers by hetero-association with IAPP mimics could become a promising approach to specifically prevent amyloid-mediated inflammation in AD, T2D, or both diseases [83].

However, there was no correlation between peripheral hIAPP and AD risk in animal and human studies [83]. In the Tg2576 AD mouse model, IAPP plasma levels were not significantly elevated at an age where the mice exhibit the glucose intolerance of pre-diabetes. Based on this negative data, it appears unlikely that peripheral IAPP cross-seeds Aβ pathology in the AD brain. However, IAPP protein is present in astrocytes in the murine brains and secreted from primary cultured astrocytes [83]. This preliminary report suggests a potential and novel association between brain-derived IAPP and AD, however, whether astrocytic-derived IAPP cross-seeds Aβ in the brain necessitates additional research.

Peripheral hIAPP serum level is not different in AD and controls [83]. Therefore, peripheral hIAPP may not be the partner in AD neuropathology, though brain-derived hIAPP could be the main in AD neuropathology. However, cerebrospinal fluid (CSF) hIAPP level is augmented in AD patients with or without T2DM [83]. Moreover, deposition of hIAPP alone or mixed with hIAPP in the temporal lobe are increased in both T2DM and late-onset AD patients [86]. High hIAPP in AD implicates its role in AD neuropathology, however, the origin of hIAPP could be from the brain, or pancreatic islet remain not fully elucidated [87]. An experimental study confirmed that local brain production of hIAPP contributes to AD neuropathology [87]. Therefore, hIAPP was detected in both brain parenchyma and CSF of AD patients [88].

**Fig. 4:**
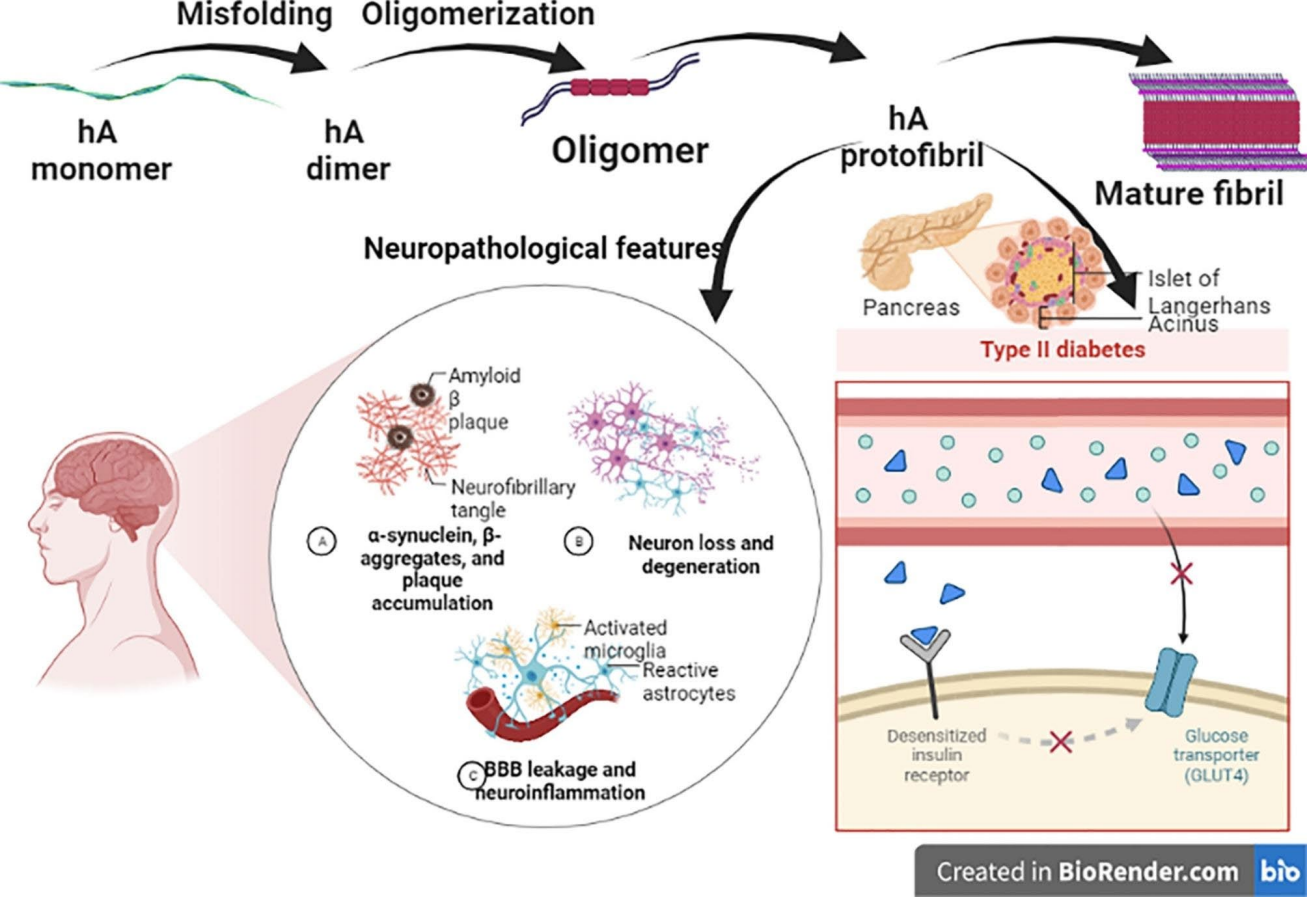
Role of human Islet amyloid polypeptide (hIAPP) in Alzheimer’s disease (AD)

Deposition of hIAPP in both T2DM and AD is due to metabolic dysfunction, microvascular injury, and failure of Aβ clearance [86]. Besides, the deposition of tau proteins and Aβ are also found in the pancreatic islet [89]. These verdicts indicate the association between T2DM and AD (Fig. 4).

In the normal state, unmodified (soluble) hIAPP crosses BBB and enhances clearance of Aβ4 to prevent the development and progression of AD neuropathology [90]. When unmodified hIAPP is subjected to oxidative stress factors, it becomes modified with subsequent loss of its protective biology. Modified hIAPP has the propensity to aggregate and be involved in AD neuropathology [90]. Schultz et al. [90] elucidated that total CSF hIAPP level was not differed in AD compared to individuals with cognitive impairments. However, total CSF hIAPP level was positively correlated with tau protein level and negatively correlated with CSF Aβ_42_ level [90]. Both hIAPP and Aβ have interacted and seeded each other to form hybrid amyloid fibrils [85]. The correlation between plasma and CSF levels of unmodified hIAPP suggests a well-functional translocation of peripherally-derived unmodified hIAPP over the BBB under normal conditions. This crossing is important for brain processes dependent on hIAPP access, such as appetite regulation and cognition [83]. Interestingly, the correlation between CSF and plasma unmodified hIAPP levels was lost in patients with AD both with and without T2DM; a result indicating either that normal BBB crossing of unmodified hIAPP is compromised or clearance of unmodified hIAPP into the CSF is dysfunctional in these patients [91]. In contrast to unmodified hIAPP, levels of total IAPP in plasma did not correlate with the same in CSF. This disassociation could be due to a limitation of the amount of IAPP that can cross the BBB [83, 91]. In particular, hIAPP is associated with a higher AD risk by different mechanisms. Circulating hIAPP affects neuronal functions independent of Aβ_42_ level even in non-diabetic status, hIAPP disrupts neurovascular injury, interacts with Aβ_42_ and acts as a seed to increase deposition of Aβ plaques [92]. In addition, hIAPP exacerbates the toxic effects of Aβ_42_ by increasing the production of ROS. Likewise, Aβ_42_ interacts, cross seed and increases the toxic effects of hIAPP [93]. Co-aggregation of hIAPP and Aβ_42_ increases tau protein hyperphosphorylation through induction of oxidative stress and inflammatory changes causing progressive synaptic loss and synaptic dysfunction [94]. IAPP colocalizes with pathological tau in AD brain. IAPP binds tau and promotes its aggregation into a more toxic strain that shows increased seeding activity and neurotoxicity in vitro. Intra-hippocampal injection of the IAPP-modified tau fibrils into the tau transgenic mice induced more severe tau pathology and cognitive deficits when compared with tau fibrils. Hence, our results indicate that IAPP cross-seeds tau and mediates the spreading of tau pathology in AD [94, 95]. Synthetic Aβ aggregates intravenously injected into hIAPP transgenic mice triggered IAPP amyloid formation, and accelerated pancreatic pathology, supporting the cross-seeding interaction between these peptides [87]. Given that both hIAPP and tau are amyloidogenic and prone to aggregate into amyloid deposits, hIAPP may interact with tau and enhance tau pathology [88, 96]. However, the factors that initiate the formation of different strains remain elusive.

A recent study involved postmortem brain tissues of AD patients together with in vitro and experimental studies illustrated that hIAPP promotes tau protein deposition [95]. In addition, peripheral hIAPP promotes the conversion of tau protein to more toxic tau fibrils [94] suggesting that hIAPP plays an assisting role in AD neuropathology. However, the assembly of Aβ_42_ is prevented by hIAPP and hIAPP analogue pramlintide which attenuate the development of AD and other neurodegenerative diseases [97]. Taken together, peripheral hIAPP plays a major role in the pathogenesis of AD, and high circulating hIAPP level increase AD risk in T2DM patients. However, there is no hard evidence for the role of brain-derived hIAPP in the pathogenesis of AD.

## Discussion

Interestingly, hIAPP undergoes tertiary structural changes and can form aggregates that deposit in the pancreatic islet causing β cells dysfunction even in the prediabetic period [98]. Both AD and T2DM share a common feature of protein deposits, hIAPP in TDM and Aβ in AD. In addition, hIAPP shares many physiochemical properties of Aβ [98]. Particularly, both Aβ and hIAPP are degraded by IDE and NEP, therefore mutation of these enzymes prevents degradation of Aβ and hIAPP with subsequent accumulation in both brain and pancreatic islets leading to AD and IR [99]. Therefore, boosting the activity of IDE and NEP may reduce the risk for the development of both AD and T2DM. The potential role of hIAPP in the development and progression of AD in T2DM was well elucidated by different studies [100, 101]. However, the novelty of the present review is to review the mechanistic role of pancreatic and brain-derived hIAPP in the induction of AD neuropathology.

The mode of toxicity by hIAPP and Aβ despite is mediated in a receptor-dependent manner. Receptor activity modifying protein 3 (RAMP3) and calcitonin receptors which are highly expressed in the brain act as a receptor for hIAPP to increase Aβ toxicity [102]. In addition, the expression of hIAPP receptors is correlated with Aβ load since these receptors are required for the effect of Aβ [102]. In this state, Aβ-induced cholinergic toxicity in rats is prevented by hIAPP antagonists which also attenuate Aβ-mediate apoptosis [103]. Aβ and hIAPP induced neuronal injury and neurotoxicity through the activation of integrin signaling [104]. Likewise, Aβ and hIAPP can increase Aβ and associated AD neuropathology by augmentation of the expression of amyloid precursor protein (APP) [105]. Therefore, Aβ neurotoxicity is mediated by the action of hIAPP. Comparable to Aβ, hIAPP can cause apoptosis and neuronal cell deaths as confirmed by in vitro study [102]. Aβ-hIAPP oligomerized mixture has three-fold more neurotoxic effects compared to hIAPP and Aβ alone [102]. In particular, Aβ-hIAPP oligomerized, hIAPP and Aβ lead to more neurotoxic effects on the hippocampal neurons [102] indicating the vulnerability of hippocampal neurons to the neurotoxic effect of hIAPP and Aβ. Notably, hIAPP and Aβ can bind neuronal and non-neuronal cell membranes leading to synaptic dysfunction through the induction of oxidative stress and mitochondrial dysfunction [106]. These verdicts proposed that hIAPP and Aβ can interact at different levels to induce neurotoxicity and neuronal injury to induce the development and progression of AD. However, not all T2DM patients develop AD in relation to high circulating hIAPP. Thus, precipitating factors in T2DM and AD could be the underlying mechanisms for the aggregation of hIAPP and increase the interaction between hIAPP and Aβ. Of note, oxidative stress and mitochondrial dysfunction are common in both T2DM and AD [56, 107]. These pathological conditions enhance aggregation and promote the interaction of hIAPP with Aβ to induce neurotoxicity [108]. As mentioned above, chaperon-mediated autophagy binds and increases the clearance of hIAPP [58] this process is dysregulated in both T2DM and AD [58, 109]. Therefore, cellular dysfunctions including oxidative stress, mitochondrial dysfunction, and dysregulated chaperon-mediated autophagy could the possible mechanism for the aggregation of hIAPP with the development of AD neuropathology in T2DM. The use of antioxidants or autophagy activators may reduce AD risk in T2DM patients [109, 110].

Interestingly, HSPG and perlecan which increase hIAPP aggregation [61] are dysregulated in T2DM and associated complications [111]. Hyperglycemia in T2DM induces significant alteration of HSPG in the endothelium and brain by increasing expression of heparanase enzyme which degrades HSPG [112]. Dysregulated HSPG in T2DM promotes neuronal injury by increasing the aggregation of hIAPP [61] leading to the development and progression of AD. It has been shown that HSPG is highly expressed in the brain mainly in Aβ deposits in transgenic mice and AD patients [113]. In AD, the expression of HSPG is altered in the brain [114]. A case-controlled study on 18 AD patients and 6 healthy controls revealed that core protein HSPG was increased in AD patients compared to the control [115]. This finding suggests that HSPG could be a potent inducer of AD neuropathology through the increasing aggregation of hIAPP. Thus, dysregulated HSPG in T2DM patients enhances hIAPP aggregation leading to the development of AD.

Furthermore, abnormal immune response to the aggregated hIAPP triggers releases of pro-inflammatory cytokines and expression of inflammatory signaling pathways that augment hIAPP-induced cytotoxicity in both T2DM [62] and AD [115]. Of note, NF-κB is involved in the pathogenesis of AD and T2DM leading to neuroinflammation and inflammatory reaction in the pancreatic islet respectively [116, 117]. NF-κB accelerates aggregation of hIAPP in mice [63]. Likewise, the NLRP3 inflammasome which is activated in both AD and T2DM [116, 117] also accelerates the aggregation of hIAPP [118]. These findings indicated that an exaggerated immune response against hIAPP induces more pancreatic islet cytotoxicity and neurotoxicity.

In addition, zinc has a protective effect against protein misfolding and aggregation of hIAPP [66]. Zinc deficiency enhances the aggregation of hIAPP and the development of injury of pancreatic β cells [66]. It has been reported that zinc level is reduced in both T2DM and AD [69, 119]. Zinc has a neuroprotective effect in normal concentration, though a higher concentration of zinc leads to neurotoxicity [119]. Zinc modulates APP function and inhibits protein phosphatase 2 A which is involved in tau protein phosphorylation [12]. However, higher zinc level promotes tau protein hyperphosphorylation and AD neuropathology [119]. Therefore, zinc has bidirectional effects that could be beneficial or detrimental to AD neuropathology. In this state, zinc dyshomeostasis in T2DM affects AD neuropathology through modulation of the aggregation of hIAPP.

Taken together, oxidative stress, mitochondrial dysfunction, chaperon-mediated autophagy, HSPG, immune response, and zinc homeostasis in T2DM could be the possible mechanisms for induction of the aggregation of hIAPP which increase AD risk.

## Effects of anti-diabetic drugs on hIAPP and AD

### Insulin sensitizing agents

It has been detected that IR is linked with mild cognitive impairment in AD patients compared to controls due to dysfunction of brain insulin signaling [120]. Administration of insulin did not reduce IR in postmortem brain tissue [120]. Consequently, overcoming IR by insulin-sensitizing agents could be more effective in reducing brain IR and AD neuropathology. Metformin is an insulin-sensitizing agent that belongs to the biguanid group used as first-line therapy in the management of T2DM and has the capability to decrease peripheral IR [121]. Metformin can reduce the production and aggregation of Aβ in the hippocampus and cerebral cortex by increasing the activity of IDE [122]. AD is regarded as type 3 diabetes mellitus (T3DM) due to the sharing similarity with T2DM and the development of brain IR [123]. Therefore, metformin may act in a double role in the prevention and treatment of both T2DM and AD. Preclinical and pilot studies propose that metformin therapy is effective in the management of AD [124]. Treatment of mice with metformin for 8 weeks amended cognitive function by inhibiting Aβ accumulation [124]. A randomized study exemplified that metformin recovers memory and cognitive functions [125]. Additionally, a modern study confirms that metformin advances cognitive and memory function reduces Aβ accumulation and links to inflammation and oxidative stress [126]. An epidemiological study on T2DM patients on diverse diabetic treatments demonstrated that long-term metformin therapy was linked with more reduction in the hazard ratio for development of AD compared to sulfonylurea [127]. As well, long-standing metformin therapy improves learning function and attention ability by increasing orbito-frontal neuronal metabolism which extremely concerned in AD [128]. This finding suggests that metformin leads to a selective effect on brain regions. Interestingly, metformin has ability to cross BBB and controls brain IR by modulating the expression of adenosine monophosphate kinase protein kinase (AMPK) which augments expression of insulin receptor substrate 1 (IRS-1) [129] leading to the inhibition the production of Aβ, tau protein phosphorylation and induction of autophagy [130]. Likewise, metformin attenuates IR development and reduces the expression of tau hyperphosphorylation [131]. Brain IR triggers the expression of tau protein kinases like glycogen synthase kinase 3 β (GSK3β) which increase tau hyperphosphorylation, misfolding and oligomerization in postsynaptic neurons [132]. IDE and GSK3β are altered in T2DM and AD. Upregulation of GSK3β and downregulation of IDE are associated with the development of AD in T2DM [133]. Brain IR may develop due to Aβ accumulation as deletion of Aβ enhances peripheral and brain insulin sensitivity [134]. These findings proposed a vicious cycle between Aβ accumulation and brain IR. Besides, chronic hyperglycemia and associated glucolipotoxicity in T2DM promote the generation of advanced glycation end-products (AGEs) which are regarded as a possible link between T2DM and late-onset AD [135]. Increasing of brain AGEs promote development of AD through activation of APP processing, deposition of Aβ and Aβ fibrilisation. Likewise, AGEs trigger the expression of AGEs receptors which are considered as possible receptors of Aβ [136]. Metformin regulates Aβ production and associated oxidative stress and mitochondrial dysfunction. As well, metformin reduces apoptotic cell deaths and increases neurogenesis [137]. The beneficial effects of metformin therapy in AD are by different mechanism including normalization of tau protein metabolism and phosphorylation, regulation of autophagy and Aβ accumulation. In virtue of its anti-inflammatory effect of metformin improves cognitive function and may attenuate the progression of AD neuropathology via induction of Aβ clearance with inhibition of tau protein phosphorylation and associated neuroinflammation [138].

Furthermore, the thiazolidinediones (TZDs), a family of peroxisome proliferator–activated receptor γ (PPARγ) activators like rosiglitazone and pioglitazone are antidiabetic agents that primarily act by improving peripheral insulin sensitivity [139]. TZDs improve pancreatic β-cell function and insulin sensitivity in diabetic animals and T2DM patients [139–141].

It has been demonstrated that hIAP formation in vivo is markedly reduced by the effects of rosiglitazone and metformin in hIAPP transgenic male mice fed a moderate-fat diet for 1 year [142]. Rosiglitazone and metformin treatment resulted in reduced β-cell secretory demand, as shown by reduced insulin secretion in response to intravenous glucose, with the maintenance of euglycemia [142]. The mean insulin response in the rosiglitazone-treated mice was unexpectedly low, due to paradoxical negative values for the acute insulin response to glucose in three mice [142]. Thus, in this case, the reduction of islet mass with rosiglitazone and metformin appears to have been appropriate for the agents’ effects to reduce the secretory stimulus to the β-cell [142]. Reduction in the proportion of pancreatic β-cell mass to islet mass was strongly correlated with increased hIAPP severity, consistent with an effect of amyloid to result in β-cell loss [142]. Therefore, treatment with both rosiglitazone and metformin, while resulting in reduced β-cell mass through reduced secretory demand, acted to preserve the remaining β-cell population through suppression of islet amyloid formation. This effect of rosiglitazone and metformin to reduce islet amyloid formation through decreased secretory demand is consistent with studies showing that increased secretory demand due to obesity and IR is linked with marked deposition in hIAPP transgenic mice, whereas decreased insulin output by disrupting β-cell glucokinase resulted in a significant reduction in islet amyloid deposition [143]. Therefore, rosiglitazone or metformin treatment significantly reduces both the prevalence and severity of islet amyloid in hIAPP transgenic mice. These effects appear to be mediated in part through the ability of these agents to alter visceral fat deposition, thus reducing secretory demand on the compromised β-cell that is capable of forming islet amyloid. Further, it would appear that both agents might have an effect beyond simply changing visceral fat, with this effect being greater with rosiglitazone [142, 143]. A systematic review on the effects of insulin sensitizer including metformin and TZDs on AD neuropathology showed these agents were effective in reducing Aβ pathology [144]. Findings from clinical trials and current safety data suggest that rosiglitazone should not be used for the treatment of AD. Application of results from trials evaluating pioglitazone in the treatment of AD is limited because of major trial limitations; therefore, it should not be recommended at this time [145]. However, a preliminary study suggested that rosiglitazone may offer a novel strategy for the treatment of cognitive decline associated with AD [146]. Relative to the placebo group, subjects receiving rosiglitazone exhibited better delayed recall and selective attention. Plasma Aβ levels were unchanged from baseline for subjects receiving rosiglitazone but declined for subjects receiving placebo, consistent with recent reports that plasma Aβ42 decreases with progression of AD [146]. No evidence of the efficacy of 2 mg or 8 mg rosiglitazone monotherapy in cognition or global function was detected in the *APOE-*Ε*4*-negative or other analysis populations [147]. These findings proposed that insulin-sensitizing agents’ metformin and TZDs could be effective in both T2DM and AD through modulation of hIAPP.

### Dipeptidyl dipeptidase inhibitors and incretin analogues

Dipeptidyl peptidase 4 (DPP4) inhibitors like sitagliptin are widely used and tolerated in the management of T2DM with minimal or no hypoglycemia as an adverse effect [148, 149]. Preclinical studies indicated that DPP4 inhibitors are effective against AD neuropathology [150, 151]. The long-term inhibition of the endogenous DPP-4 enzymes with sitagliptin can meaningfully delay some forms of AD pathology, including Aβ deposition, when administered early in the disease course of a transgenic mouse model of AD [150]. DPP4 inhibitor saxagliptin which increases the level of glucagon-like peptide-1 (GLP-1) and ameliorates T2DM, has become a valuable candidate as a disease-modifying agent in the treatment of AD. In addition, endogenous GLP-1 levels decrease Aβ peptide and tau phosphorylation in the AD rat models [151]. Furthermore, GLP1 receptor agonists have also been shown as possessing neuroprotective effects in AD, which seem to improve nearly all neuropathological features as well as cognitive functions of AD [152]. For example, NFTs, amyloid plaques, and neuro-inflammations in the hippocampus have been reduced in AD model mice [153]. It has been shown that a GLP1 receptor agonist also prevents synaptic damage induced by Aβ accumulation, which supports spatial memory by affecting the phosphoinositide-3 kinase (PI3K)-AKT pathway [154]. Targeting DPP4 inhibitors that is involved in the GLP1 signaling has been considered as a promising therapeutic model for AD [155]. Furthermore, the mammalian/mechanistic target of rapamycin (mTOR) has been considered as a center that integrates multiple signaling cascades including the GLP1 receptor signaling, which may also be involved in the progression of AD [155]. Moreover, treatment with DPP4 inhibitor vildagliptin restored the islet topography by modulating the expression of hIAP [156]. Notably, sitagliptin preserves pancreatic β-cell mass and function and enhances insulin sensitivity in the hIAP rat model of T2DM [157]. Therefore, DPP4 inhibitors and GLP-1 agonists attenuate AD in T2DM by inhibiting expression and deposition of hIAP.

## Conclusion

hIAPP is one of the main secretory products of pancreatic β cells in the islet of Langerhans. hIAPP has different physiological functions including inhibiting gastric empty, regulation of satiety and inhibiting the release of insulin and glucagon. T2DM is an endocrine disorder due to relative insulin insufficiency and IR, characterized by hyperglycemia and cardiometabolic complications. Interestingly, hIAPP has structural similarity with Aβ and can involve in the pathogenesis of T2DM and AD. The underlying causative factors that convert soluble hIAPP oligomers to toxic non-soluble fibrils are largely unknown. IR, aging and low β cell mass increases the expression of hIAPP which binds the cell membrane leading to the aberrant release of Ca^2+^ and activation of the proteolytic enzyme leading to series of events causing loss of β cells. hIAPP-induced dysfunction of pancreatic β cells through induction of oxidative stress and ER stress. Zinc deficiency and exaggerated immune response against hIAPP aggregation trigger more injury of pancreatic β cells. Peripheral hIAPP plays a major role in the pathogenesis of AD, and high circulating hIAPP level increase AD risk in T2DM patients. However, there is no hard evidence for the role of brain-derived hIAPP in the pathogenesis of AD. In sum, oxidative stress, mitochondrial dysfunction, chaperon-mediated autophagy, HSPG, immune response and zinc homeostasis in T2DM could be the possible mechanisms for induction the aggregation of hIAPP which increase AD risk. In addition, DPP4 inhibitors and GLP-1 agonists attenuate AD in T2DM by inhibiting the expression and deposition of hIAP. Despite these evidences the underlying mechanism linking pancreatic islet hIAPP in the development of AD need to be elucidated. Therefore, retrospective and prospective studies are recommended in this state.

## Data Availability

Not applicable.

## List of Abbreviations

hIAPP: Human Islet amyloid polypeptide
T2DM: Type 2 diabetes mellitus
Aβ: Amyloid beta
AD: Alzheimer’s disease
RAS: Renin-angiotensin system
CGRP: Calcitonin gene-related peptide
IR: Insulin resistance
IDE: Insulin-degrading enzyme
NEP: Neprilysin
RAMPs: Receptor activity modifying proteins
ROS: Reactive oxygen species
Hsp70: Heat shock protein 70
ER: Endoplasmic reticulum
HSPG: Heparan sulfate proteoglycan
NF-κB: Nuclear factor kappa B
NFTs: Neurofibrillary tangles
BBB: Blood-brain barrier
CSF: Cerebrospinal fluid
NLRP3: Node like receptor pyrin 3
RAMP3: Receptor activity modifying protein 3
APP: Amyloid precursor protein
T3DM: Type 3 diabetes mellitus
AMPK: Adenosine monophosphate kinase protein kinase
IRS-1: Insulin receptor substrate 1
GSK3β: Glycogen synthase kinase 3 β
AGEs: Advanced glycation end-products
TZDs: Thiazolidinediones
PPARγ: Peroxisome proliferator–activated receptor γ
DPP4: Dipeptidyl peptidase 4
GLP-1: Glucagon-like peptide-1
PI3K: Phosphoinositide-3 kinase
